# Paucibacillary Pleural Tuberculosis Presenting as Subpleural Nodules: A Diagnostic Challenge With Negative PCR and Smear Tests

**DOI:** 10.1002/ccr3.72205

**Published:** 2026-03-08

**Authors:** Omar Al Ayoubi, Mohammad Alaa Aldakak, Bassel Ibrahim, Raneem Ahmad, Youness Souleiman

**Affiliations:** ^1^ Faculty of Medicine Damascus University Damascus Syrian Arab Republic; ^2^ National University Hospital, Damascus University Damascus Syrian Arab Republic

**Keywords:** case report, paucibacillary disease, pleural tuberculosis, subpleural nodules, thoracoscopic biopsy

## Abstract

Pleural tuberculosis is a common manifestation of extrapulmonary tuberculosis; however, its diagnosis remains challenging in paucibacillary disease, where clinical presentation may be atypical and microbiological tests frequently yield negative results. We report the case of a 27‐year‐old Syrian male who presented with a one‐year history of left‐sided pleuritic chest pain and unintentional weight loss. Imaging studies revealed left‐sided pleural‐based subpleural nodules with mild metabolic activity. Repeated sputum acid‐fast bacilli smears, polymerase chain reaction testing for *Mycobacterium tuberculosis* , and bronchoscopy were all negative. Due to persistent clinical suspicion, thoracoscopic exploration was performed, revealing multiple subpleural nodules on both the parietal and visceral pleura. Histopathological examination demonstrated necrotizing granulomatous inflammation with caseous material, consistent with pleural tuberculosis. The patient was treated with a standard six‐month antituberculous regimen and showed favorable clinical recovery. Pleural tuberculosis represents a diagnostic challenge due to its frequent paucibacillary nature and nonspecific clinical presentation, which often results in low sensitivity of conventional microbiological and molecular tests such as direct smears using Ziehl–Neelsen and Auramine staining. In this case, prolonged pleuritic chest pain with minimal systemic symptoms and repeatedly negative sputum smears and PCR delayed microbiological confirmation. Definitive diagnosis was achieved only through thoracoscopic pleural biopsy and histopathological examination. This highlights the limitations of noninvasive investigations in pleural TB and underscores the importance of early escalation to pleural biopsy when clinical suspicion persists despite inconclusive results. This case highlights pleural tuberculosis presenting as subpleural nodules as a diagnostic challenge in the setting of negative microbiological tests. Maintaining a high index of suspicion and early escalation to tissue diagnosis are essential to ensure timely treatment and prevent misdiagnosis.

## Key Clinical Message

Pleural tuberculosis may present as isolated subpleural nodules, with repeatedly negative sputum smears and PCR results due to the paucibacillary nature of the disease. When clinical suspicion persists despite negative microbiological testing, early pleural biopsy should be considered to obtain diagnostic tissue and promptly initiate appropriate therapy.

## 1 Introduction

Tuberculosis (TB) remains a significant global public health concern, with the global incidence rate of TB approximately 130 cases per 100,000 persons [1, 2]. Extrapulmonary tuberculosis (EPTB) is defined as infection by *Mycobacterium tuberculosis* (MTB) involving any organ other than the lungs. Globally, EPTB accounts for about 16% of the 7.5 million notified TB cases, with reported proportions ranging from 8% in the Western Pacific Region to 24% in the Eastern Mediterranean Region. The pleura is the second most common site of EPTB after the Peritoneum, representing 10%–15% of the extrapulmonary cases globally reported [3, 4]. Diagnosing TB can be especially difficult when the clinical presentation is atypical, symptoms are non‐specific, or the bacterial load is low, as seen in paucibacillary disease. In these situations, standard diagnostic methods, such as acid‐fast bacilli (AFB) smear microscopy and nucleic acid amplification tests (NAATs), often produce negative results, which can result in missed diagnoses or significant delays in starting treatment [5, 6]. These diagnostic delays are clinically important, as they have been linked to worse outcomes, such as treatment failure, loss to follow‐up, and an increased risk of mortality [5]. Here, we present a case of a 27‐year‐old male with left‐sided chest pain, weight loss, and negative sputum tests, but imaging and histopathology suggested pleural tuberculosis. Despite negative microbiological results, granulomatous inflammation consistent with TB was identified, highlighting the challenges of diagnosing paucibacillary TB.

## 2 Case Presentation

### 2.1 Clinical Presentation

A 27‐year‐old Syrian male presented to our thoracic department with a one‐year history of left‐sided chest pain, exacerbated by coughing and deep inspiration. The pain started after his release from prison, and the patient attributed it to a previous left clavicular fracture and fixation. He reported an unintentional weight loss of 5 kg over the past year, with no history of night sweats or hemoptysis. He had no significant past medical history apart from a left clavicular fracture treated with a plate and a right femoral fracture treated with fixation following a motor vehicle accident. He was a smoker with a 10‐pack‐year history and reported occasional alcohol consumption.

On clinical examination, his vital signs were stable: blood pressure 130/70 mmHg, heart rate 75 beats per minute, temperature 37°C, oxygen saturation 95%, and respiratory rate 24 breaths per minute. There were no signs of pallor, jaundice, lymphadenopathy, or clubbing, and chest examination revealed clear, symmetrical breath sounds.

### 2.2 Diagnostic Work‐Up

**FIGURE 1:**
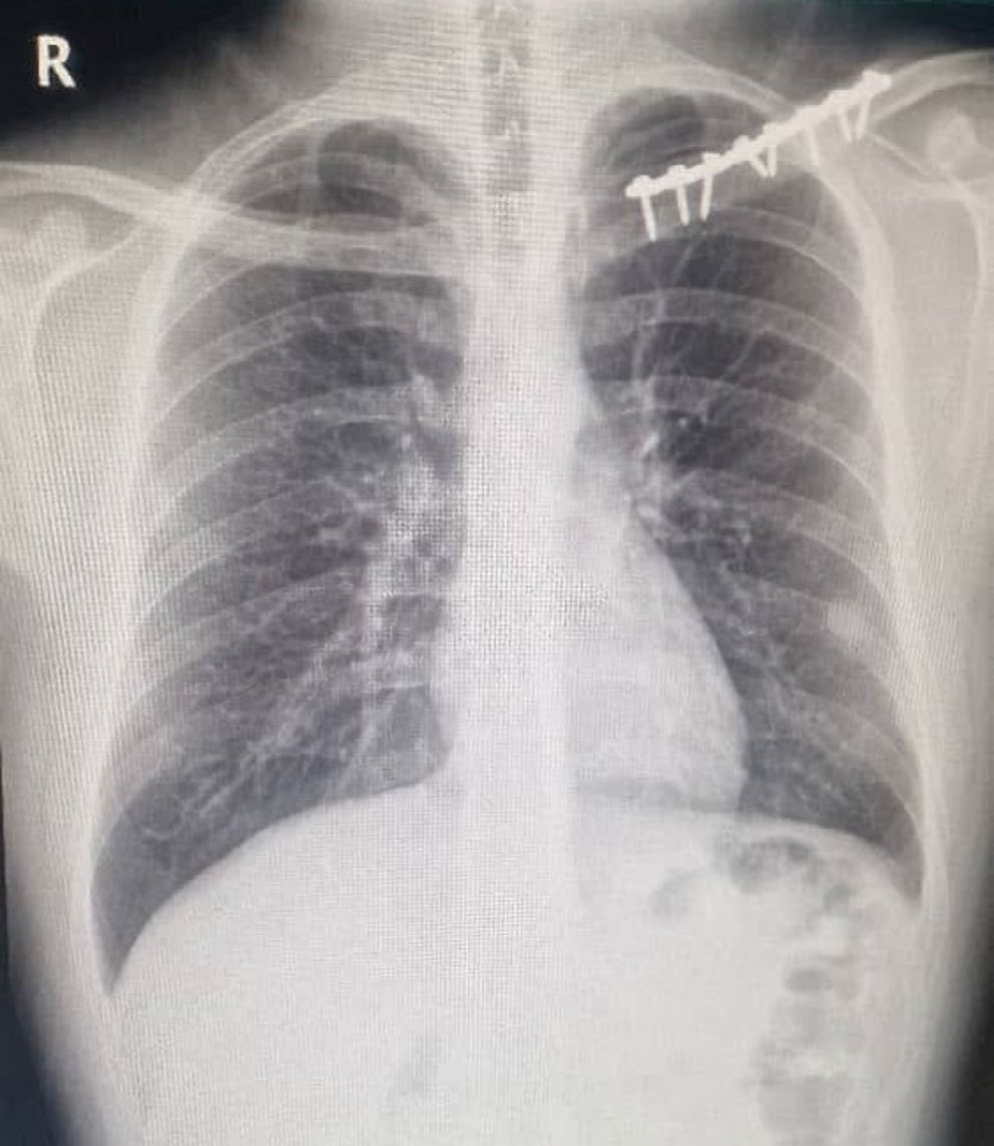
Posteroanterior chest radiograph showing left clavicular fixation hardware with otherwise clear lung fields and a subtle pleural‐based opacity along the lateral aspect of the left lower hemithorax.

**FIGURE 2:**
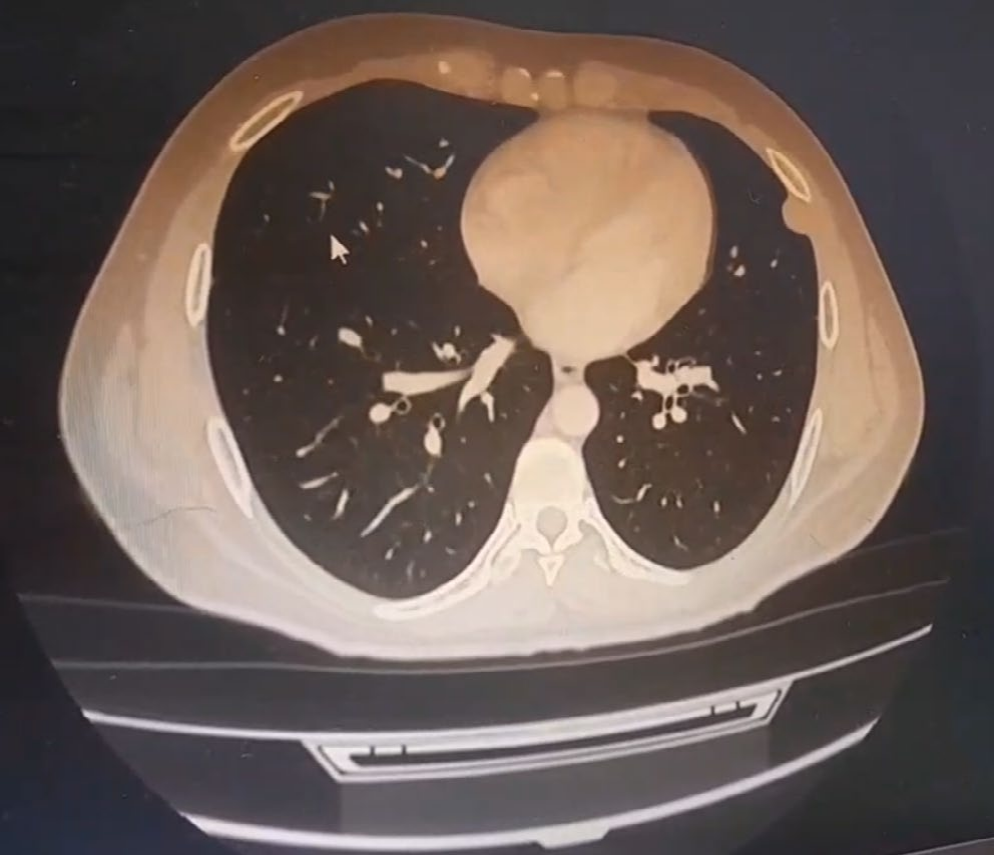
Axial chest CT (lung window) demonstrating a left pleural‐based subpleural lesion in the lateral segment of the lower lobe abutting the chest wall, without associated parenchymal consolidation.

**FIGURE 3:**
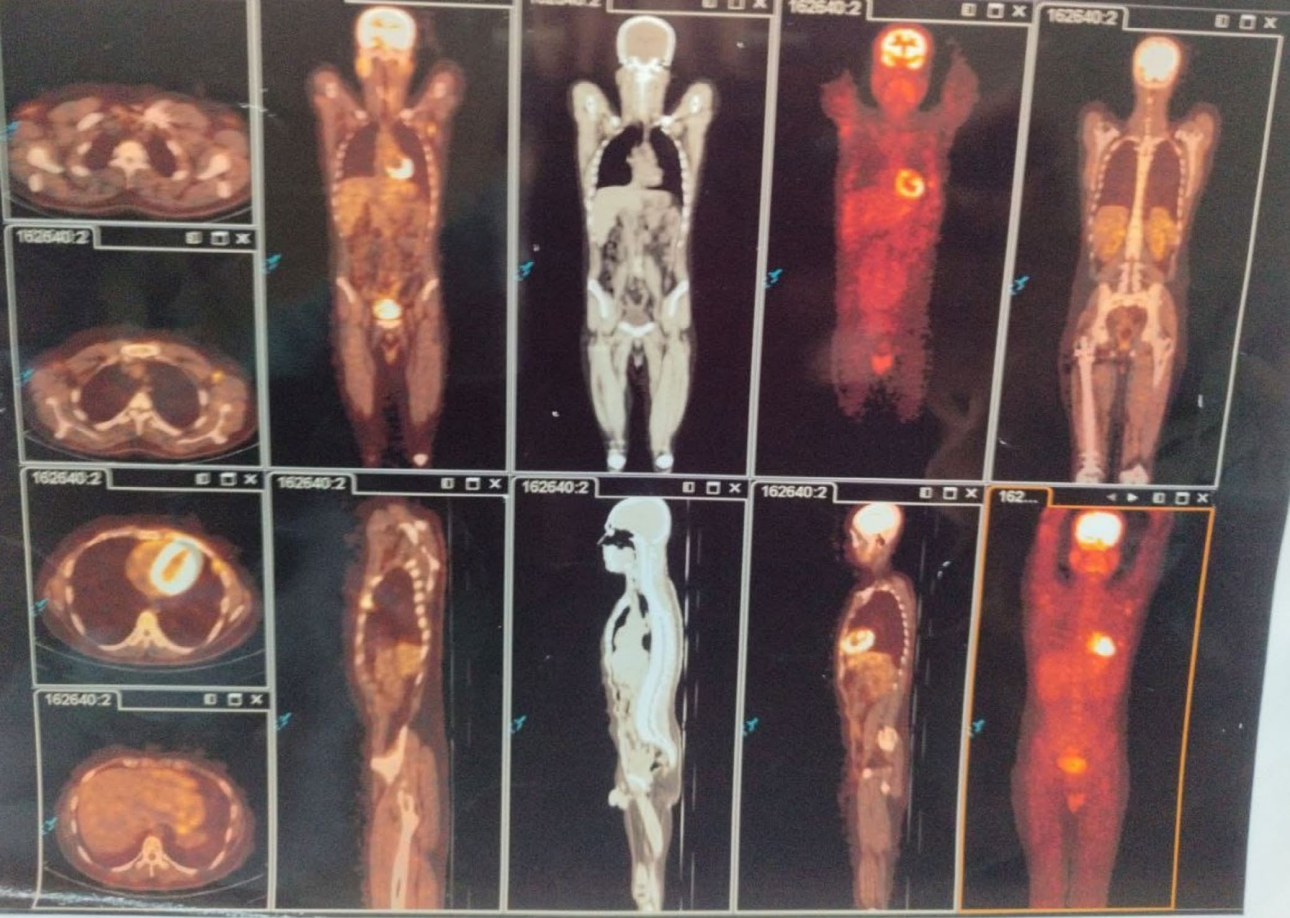
Whole‐body FDG PET/CT showing mild metabolic activity (SUV < 3) in the left subpleural pleural‐based lesion and small bilateral axillary lymph nodes, with no abnormal uptake in the cervical, mediastinal, abdominal, pelvic, or inguinal regions.

Following the clinical assessment, a chest radiograph (X‐ray) was obtained, which demonstrated a left‐sided pleural‐based opacity adjacent to the chest wall (Figure 1). To further characterize this lesion and assess its relationship to the previous clavicular hardware, a chest computed tomography (CT) scan was performed, confirming a left‐sided pleural‐based density abutting the chest wall (Figure 2). This finding prompted further evaluation with positron emission tomography (PET), which showed left subpleural lymph nodes, the largest measuring 22 mm, with mild metabolic activity (SUV < 3). Bilateral axillary lymph nodes demonstrated similar low‐grade uptake, while no pathological involvement was observed in the cervical, mediastinal, pelvic, or inguinal lymph nodes (Figure 3).

Based on these imaging findings, the patient underwent additional diagnostic workup. Sputum analysis was negative for AFB on three separate Ziehl–Neelsen stains. A tuberculin skin test (TST) showed a 7‐mm induration, suggestive of prior exposure to TB despite the negative microbiological studies. Flexible bronchoscopy revealed patent airways without endobronchial lesions or signs of active infection, and a polymerase chain reaction (PCR) test for MTB was negative.

### 2.3 Invasive Procedures and Histopathology

**FIGURE 4:**
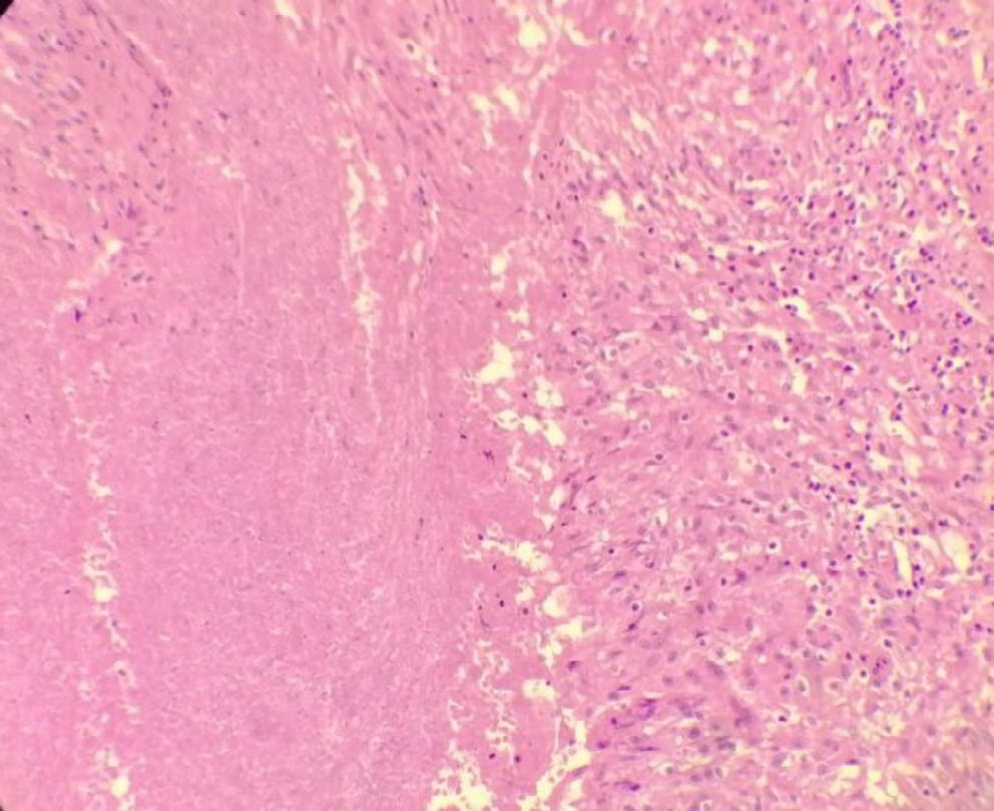
Histopathological section of an excised pleural nodule showing central amorphous caseous necrosis surrounded by granulomatous inflammation composed of epithelioid histiocytes and lymphocytes, consistent with necrotizing granulomatous inflammation (hematoxylin and eosin stain).

**FIGURE 5:**
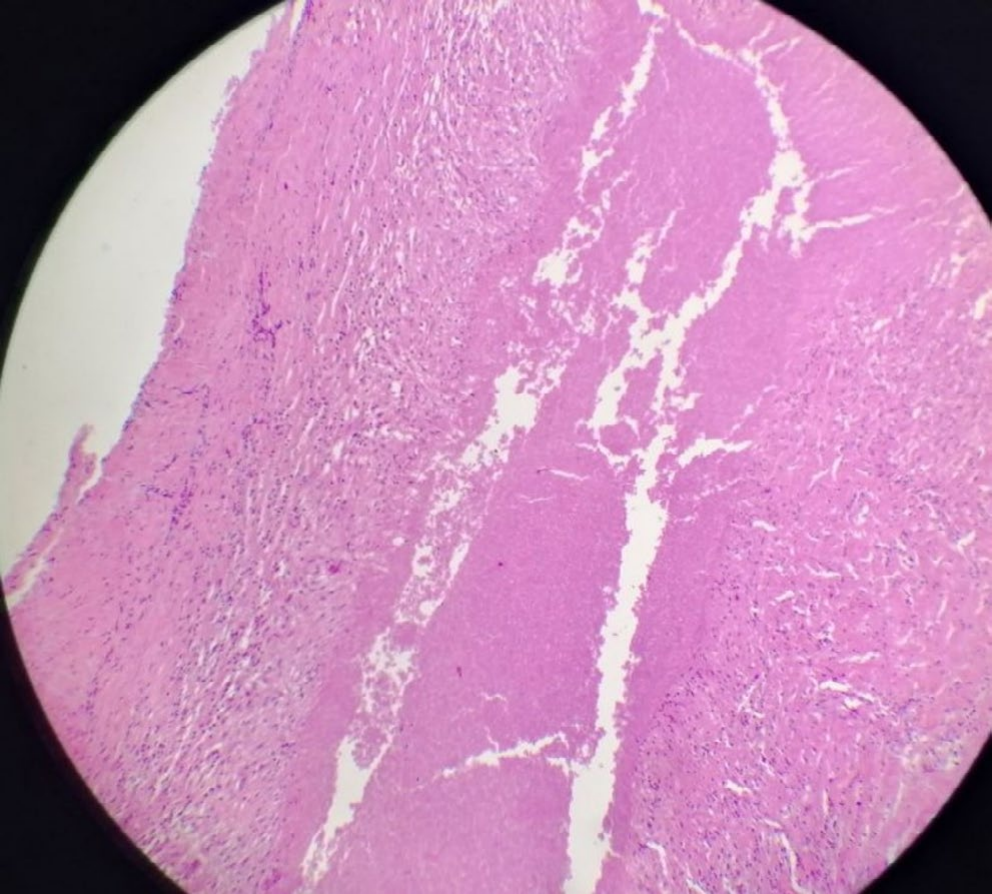
Histopathological section from another excised pleural nodule demonstrating extensive caseous necrosis with surrounding granulomatous inflammation, in keeping with tuberculous etiology (hematoxylin and eosin stain).

Given the persistent clinical suspicion and inconclusive noninvasive investigations, percutaneous image‐guided (CT/Ultrasound) pleural biopsy was considered but not performed. The lesions were small, strictly subpleural, and closely adherent to the chest wall and clavicular hardware, raising concerns about sampling failure and an increased risk of complications (e.g., pneumothorax). Therefore, diagnostic thoracoscopic exploration was preferred, allowing direct visual inspection and removal of all visible subpleural nodules for histology, and a higher diagnostic yield. Two thoracoscopic ports were inserted to access the left pleural cavity, where subpleural nodules were identified on both the parietal and visceral pleura. All visible subpleural nodules on the parietal and visceral pleura were completely excised thoracoscopically and submitted for histopathological examination, and a 32 French thoracostomy tube was placed for drainage. Histopathological examination revealed three fragments measuring 2.0 × 1.3 × 0.9 cm, 1.0 × 1.0 × 0.7 cm, and 0.7 cm in greatest dimension, respectively. The fragments contained caseous material consistent with necrotizing granulomatous inflammation, highly suggestive of TB (Figures 4 and 5).

### 2.4 Treatment and Outcome

Postoperatively, the patient had an uneventful recovery with no respiratory or systemic complications, and the chest tube was removed after adequate lung expansion and cessation of drainage. He was subsequently discharged home in good general condition with stable vital signs and no residual chest pain, and was commenced on a standard 6‐month anti‐tuberculous regimen under the supervision of the infectious diseases team.

## 3 Discussion

Tuberculosis continues to rise in many developing regions, posing a substantial public health challenge and demanding diagnostic tools that are both highly sensitive and specific for early detection of MTB [7]. Among its extrapulmonary forms, pleural tuberculosis represents a distinct clinical entity [3, 4]. Pleural tuberculosis is defined as the presence of MTB in pleural samples, demonstrated either by smear microscopy using Ziehl–Neelsen staining, by culture of pleural fluid, or by examination of pleural biopsy tissue, together with compatible clinical or radiological findings [8].

Pleural tuberculosis may arise as a primary infection or result from reactivation of latent disease [8]. The pathogenesis is driven by a delayed hypersensitivity response to mycobacterial antigens within the pleural space. Even a limited number of bacilli trigger a vigorous T‐cell–mediated inflammatory cascade, stimulating macrophages and leading to granuloma formation. This immune activity increases vascular permeability and promotes exudative effusion formation [9]. Clinically, pleural TB is seen more often in younger patients, while presentation at an older age is typical in settings where reactivation is the main pattern of disease, such as in nonendemic countries [10]. In our case, the patient was a 27‐year‐old Syrian man, an age distribution that is consistent with reports of pleural tuberculosis occurring more commonly in younger individuals in primary‐infection settings. His recent incarceration likely increased his exposure risk and thus favors recent infection rather than reactivation.

A pleural‐based/subpleural lesion with chronic pleuritic pain and low PET uptake raises several possibilities, including primary pleural malignancy (e.g., mesothelioma), pleural metastasis or peripheral lung cancer with pleural invasion, and pleural lymphoma. Infectious and inflammatory causes such as tuberculous pleuritis, fungal granulomas, empyema with organizing pleuritis, and sarcoidosis should also be considered [11, 12]. In this case, the combination of indolent symptoms, low metabolic activity on PET, repeatedly negative noninvasive microbiology, and definitive histology showing necrotizing granulomas supports paucibacillary pleural tuberculosis as the final diagnosis.

TB pleuritis should be considered in any patient presenting with unilateral pleural effusion, regardless of size [10]. Symptoms usually evolve over a short time frame and may include fever (75%), pleuritic chest pain (50%–75%), and a dry cough (70%–75%). Systemic manifestations such as night sweats, chills, fatigue, dyspnea, and weight loss are also frequently observed [10]. In contrast to the classical acute presentation of pleural tuberculosis, our patient experienced a prolonged course of left‐sided chest pain lasting one year, which worsened with coughing and deep inspiration. Notably, he did not report fever, night sweats, or hemoptysis, and his systemic symptoms were limited to gradual weight loss. This atypical presentation highlights the diagnostic challenge of paucibacillary pleural TB, as standard clinical features may be absent or subtle, potentially delaying recognition and treatment.

Most forms of EPTB are paucibacillary, meaning that only a small number of MTB bacilli are present [13]. This characteristic markedly limits the sensitivity of conventional diagnostic tests [13]. In pleural tuberculosis, for instance, microbiological and molecular assays performed on pleural fluid often demonstrate low sensitivity, with direct smears using Ziehl–Neelsen or Auramine staining detecting fewer than 10% of cases and mycobacterial culture confirming less than 30% [14]. Combining pleural biopsy with these methods can improve diagnostic yield; however, this approach is invasive and may not be feasible in resource‐limited settings [15, 16, 17]. Other indirect techniques, including biochemical markers such as adenosine deaminase (ADA) and interferon‐gamma (IFN‐γ), as well as immunological tests like the TST and interferon‐gamma release assays (IGRAs), provide only moderate diagnostic accuracy [14]. Consequently, pleural tuberculosis is frequently diagnosed based primarily on clinical suspicion, which increases the risk of misdiagnosis. This can lead to patients either receiving unnecessary antituberculosis therapy or remaining undertreated, underscoring the persistent need for more reliable and accessible diagnostic strategies in pleural TB [13, 14]. Our case illustrates these diagnostic limitations: despite three negative sputum AFB smears and a negative Polymerase Chain Reaction (PCR), microbiological confirmation was not achieved, and the TST was only borderline (7 mm), reflecting the limited sensitivity of noninvasive assays. Given the chronic symptom course and inconclusive noninvasive workup, thoracoscopic excision provided definitive histology (necrotizing granulomas) and was instrumental in establishing the diagnosis, supporting the role of pleural biopsy when less invasive tests fail [15, 16].

Management of extrapulmonary TB generally follows the standard six‐month drug regimen used for pulmonary disease, consisting of a two‐month intensive phase with rifampicin, isoniazid, pyrazinamide, and Ethambutol (RHZE), followed by four months of rifampicin and isoniazid [10]. Because pleural effusions usually reflect paucibacillary disease, shorter treatment courses have been explored and appear to be associated with low relapse rates [18]. In addition, the reduced bacillary burden implies a lower risk of spontaneous resistance, making current multidrug therapy sufficient to overcome single‐agent resistance when it occurs [10]. Consistent with current recommendations, the patient was treated with the standard six‐month multidrug regimen and demonstrated an uneventful recovery, supporting evidence that paucibacillary pleural TB responds well to conventional therapy and carries a low risk of treatment failure or relapse.

## 4 Conclusion

This case underscores the diagnostic challenge and clinical importance of paucibacillary pleural tuberculosis presenting as subpleural nodules: despite negative sputum smears and PCR, imaging and clinical context may mask an underlying mycobacterial process. Clinicians should maintain a high index of suspicion for TB in patients with pleural‐based lesions—particularly when epidemiologic risk factors are present—and escalate promptly from noninvasive testing to tissue sampling when results remain inconclusive. This case also highlights the urgent need for more sensitive, accessible diagnostics and structured follow‐up to ensure treatment response and detect relapse.

## Author Contributions

**Omar Al Ayoubi:** conceptualization, validation, writing – original draft, writing – review and editing. **Mohammad Alaa Aldakak:** investigation, visualization, writing – review and editing. **Bassel Ibrahim:** methodology, resources, writing – review and editing. **Raneem Ahmad:** investigation, methodology, writing – review and editing. **Youness Souleiman:** investigation, methodology, supervision, writing – review and editing.

## Funding

The authors have nothing to report.

## Ethics Statement

The authors have nothing to report.

## Consent

Written informed consent was obtained from the patient for publication and any accompanying images. A copy of the written consent is available for review by the Editor‐in‐Chief of this journal on request.

## Conflicts of Interest

The authors declare no conflicts of interest.

## Data Availability

Data available on request from the authors.
